# Numerical Investigation of Intra-abdominal Pressure Effects on Spinal Loads and Load-Sharing in Forward Flexion

**DOI:** 10.3389/fbioe.2019.00428

**Published:** 2019-12-17

**Authors:** Tao Liu, Kinda Khalaf, Samer Adeeb, Marwan El-Rich

**Affiliations:** ^1^Department of Mechanical Engineering, Khalifa University, Abu Dhabi, United Arab Emirates; ^2^Department of Civil and Environmental Engineering, University of Alberta, Edmonton, AB, Canada; ^3^Department of Biomedical Engineering, Khalifa University, Abu Dhabi, United Arab Emirates

**Keywords:** intra-abdominal pressure, finite element model, musculoskeletal model, spinal load, load sharing

## Abstract

The intra-abdominal pressure (IAP), which generates extensor torque and unloads the spine, is often neglected in most of the numerical studies that use musculoskeletal (MSK) or finite element (FE) spine models. Hence, the spinal loads predicted by these models may not be realistic. In this work, we quantified the effects of IAP variation in forward flexion on spinal loads and load-sharing using a novel computational tool that combines a MSK model of the trunk with a FE model of the ligamentous lumbosacral spine. The MSK model predicted the trunk muscle and reaction forces at the T12-L1 junction, with or without the IAP, which served as input in the FE model to investigate the effects of IAP on spinal loads and load-sharing. The findings confirm the unloading role of the IAP, especially at large flexion angles. Inclusion of the IAP reduced global muscle forces and disc loads, as well as the intradiscal pressure (IDP). The reduction in disc loads was compensated for by an increase in ligament forces. The IDP, as well as the strain of the annular fibers were more sensitive to the IAP at the upper levels of the spine. Including the IAP also increased the ligaments' load-sharing which reduced the role of the disc in resisting internal forces. These results are valuable for more accurate spinal computational studies, particularly toward clinical applications as well as the design of disc implants.

## Introduction

Quantifying the contribution of the active and passive components of the human trunk during various daily, occupational, or athletic activities is essential for the design of effective spinal fixation systems, and would greatly benefit research and clinical stakeholders in the field of spinal biomechanics. Intra-abdominal pressure (IAP), considered as the most likely factor to influence lumbar spinal mechanics, has been continuously investigated under static and dynamic lifting conditions for many decades now (Davis, 1956; Bartelink, 1957; Davis and Troup, 1964; Andersson et al., 1976; McGill et al., 1990; Marras and Mirka, 1996; Hagins et al., 2004). Most of the existing studies advocate that the IAP produces an extensor torque (Bartelink, 1957; Morris et al., 1961), which reduces the spinal loads and back muscle activity, hence influencing the overall loading scenarios and stability of the lumbar spine (Daggfeldt and Thorstensson, 1997, 2003; Cholewicki and Reeves, 2004). This mechanism has also served as a solution to the existing paradox in biomechanical models where the predicted spinal loads exceeded the tissue-tolerance limits during weight lifting tasks (Chaffin, 1969). Abdominal belts have therefore been prescribed therapeutically to increase the IAP and unload the spine (Harman et al., 1989; Lander et al., 1992).

On the other hand, some experimental studies have questioned the unloading role of the IAP. Nachemson et al. (1986) found that an increase in the IDP is associated with a concurrent increase in the IAP during Valsalva maneuvers. It has also been reported that trunk muscle contraction is coupled with the generation of IAP (Cholewicki et al., 2002), where EMG activity of 12 trunk muscles increased due to the elevated IAP (Cholewicki et al., 1999). McGill and Norman (1987) and McGill et al. (1990) concluded that the IAP-generated extensor moment is compensated by the flexor moment due to the co-contraction of the abdominal muscles associated with the elevated IAP. In addition, the cross-section area of the diaphragm and the moment arm of the net IAP have been considered as the reason for overestimating the extensor moment produced by the IAP (McGill and Norman, 1987; McGill, 1993).

Uncertainty about the pattern of abdominal muscle coactivity, along with elevated IAP, have hence led to the controversy surrounding the unloading role of the IAP (Arjmand and Shirazi-Adl, 2006a; Stokes et al., 2010). Although, some studies suggested that the normal physiological role of the IAP cannot be adequately explored in contrived experiments, such as the Valsalva maneuver or maximum voluntary strength exertions (Arjmand and Shirazi-Adl, 2006a; Stokes et al., 2010).

Due to the inherent complexity of the spine and its structural components, both morphologically and mechanically, numerous musculoskeletal (MSK) rigid body models, analytical and computational models, have emerged as effective tools for the assessment of the relationship between the elevated IAP, and trunk spinal load and stability. Stokes et al. (2010) confirmed the unloading role of the IAP using a biomechanical model with detailed abdominal wall structure and muscle paths. Later, they revealed that pressurization of the abdomen increases lumbar spine stability, although the degree of spinal stability was not significantly affected by selective activation of either transversus abdominis or oblique muscles (Stokes et al., 2011). The computational studies conducted by Arjmand and Shirazi-Adl (2006a) and Park et al. (2013) revealed that IAP reduced the spinal joint forces during weight bearing standing position if no abdominal muscle co-activation is considered. They also demonstrated that the unloading and stabilizing action of IAP is both posture and task specific (Arjmand and Shirazi-Adl, 2006a). The shared limitation of the aforementioned studies is that all of them used a prescribed IAP when quantifying its effects on spinal loads.

More recently, Arshad et al. (2016) explored the effects of the IAP and spinal rhythm on the spinal loads in flexion using AnyBody (AnyBody Technology, Aalborg, Denmark), where IAP could be increased based on the optimization of the total muscle stress. While previous experimental/computational investigations of the IAP effects on muscle forces (Hodges et al., 2001; Arshad et al., 2016), on spinal loads (Daggfeldt and Thorstensson, 2003; Arshad et al., 2016), and on spinal stiffness (Hodges et al., 2005) have greatly contributed to spinal biomechanics, the influence of the IAP on the IDP and spinal load-sharing remain undetermined during static flexion. This knowledge is critical for various clinical applications, including informing the design of disc implants, and shedding more light on the elusive pathophysiology of low back pain and other spinal disorders. The current research, thus, aims to first delineate the modeling of the IAP in a MSK model, and secondly to quantify the effects of the IAP on muscle forces, IDP, and spinal load-sharing in the lumbosacral spine during forward flexion. This is accomplished using our combined MSK and FE modeling methodology, previously validated and published (Liu et al., 2018).

## Materials and Methods

### Musculoskeletal Model

An AnyBody MSK model (Ver. 6.0, AnyBody Technology, Aalborg, Denmark, model version 1.63) was developed and used to simulate the musculoskeletal biomechanics of a typical male of 70 kg weight and 168 cm height subjected to 60° forward flexion, with and without IAP. The model is composed of the skull, cervical region, upper arms, rigid thorax (T1–T12 as a single segment) and five rigid lumbar vertebrae (L1–L5) together with the pelvis and sacrum. The Anterior Longitudinal Ligament (ALL), Posterior Longitudinal Ligament (PLL), Intertransverse Ligament (ITL), Ligamentum Flavum (LF), Supraspinous Ligament (SSL), and Interspinous Ligament (ISL) and Capsular Ligament (CL) were all incorporated in the model and modified to match the corresponding properties in our validated published FE model (Liu et al., 2018). The ligament forces were set to zero in the neutral standing position. The facet joint contacts were also activated during simulation.

**Figure 1:**
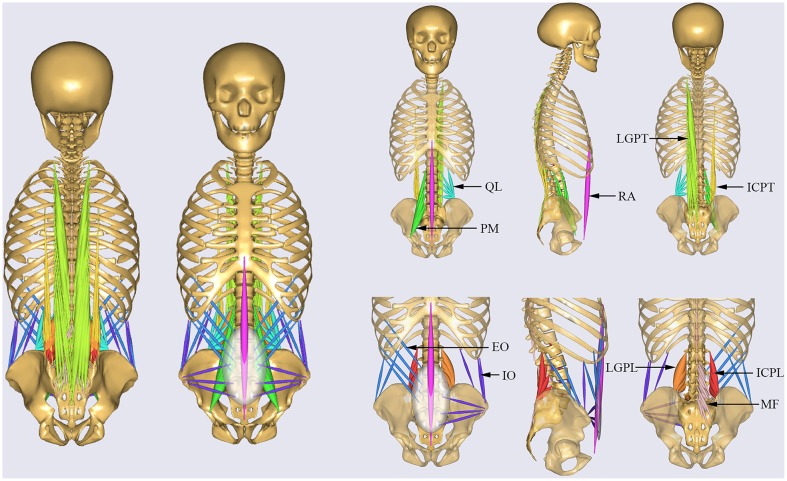
Musculature of the MSK model. Global muscles: RA, rectus abdominis; IO, internal oblique; EO, external oblique; ICPT, iliocostalis lumborum pars thoracic; LGPT, longisimus thoracis pars thoracic. Local muscles: ICPL, iliocostalis lumborum pars lumborum; LGPL, longisimus thoracis pars lumborum; PM, psoas major; MF, multifidus; QL, quadratus lumborum.

All muscles in the MSK model were simulated by one dimension elements (de Zee et al., 2007), which can resist only tensile forces. The default tensile strength for individual muscles (Ikai and Fukunaga, 1968; Arshad et al., 2017) was adopted from literature. The trunk muscles were divided into two groups (El-Rich et al., 2004), specifically global muscles and local muscles (Figure 1). The global muscles included: one rectus abdominis (RA), 12 internal oblique (IO), 12 external oblique (EO), 16 iliocostalis lumborum pars thoracic (ICPT), 24 longisimus thoracis pars thoracic (LGPT). The local muscles included: eight iliocostalis lumborum pars lumborum (ICPL), 10 longisimus thoracis pars lumborum (LGPL), 22 psoas major (PM), 38 multifidus (MF), and 10 quadratus lumborum muscle fascicles (QL) (Arshad et al., 2016). The corresponding cross section area and strength are shown in Table 1.

**Table 1:** Muscle physiological cross-sectional area (PCSA, mm^2^) for each side of the spine at different insertion levels and maximum muscle stress for each individual muscle group (in parentheses, MPa).

**Local muscles**	**ICPL(0.846)**	**LGPL(0.846)**	**PM(0.846)**	**MF(0.846)**	**QL(0.846)**
T12	–	–	211	–	128
L1	108	79	272	216	88
L2	154	91	262	314	80
L3	182	103	364	249	75
L4	189	110	239	410	70
L5	–	116	115	218	–
**Global muscles**	**ICPT(0.846)**	**LGPT(0.846)**	**IO(0.846)**	**EO (0.846)**	**RA(2.21)**
Thorax	548	1,109	624	624	260

An optimization algorithm in AnyBody based on muscle recruitment criterion was employed to calculate the load distribution among the various muscle groups. The objective function (1) used in the muscle recruitment optimization routine was to minimize the sum of the square of the ratios of muscle force to muscle strength (de Zee et al., 2007).

(1) $$\begin{array}{r} G=\overset{n}{\underset{i}{\sum }}{\left(\frac{f_{i}}{N_{i}}\right)}^{2} \end{array}$$

Where: *f*_*i*_ is the force in muscle *i*, *N*_*i*_ is the strength of muscle *i*, *n* is the total number of muscles.

**Figure 2:**
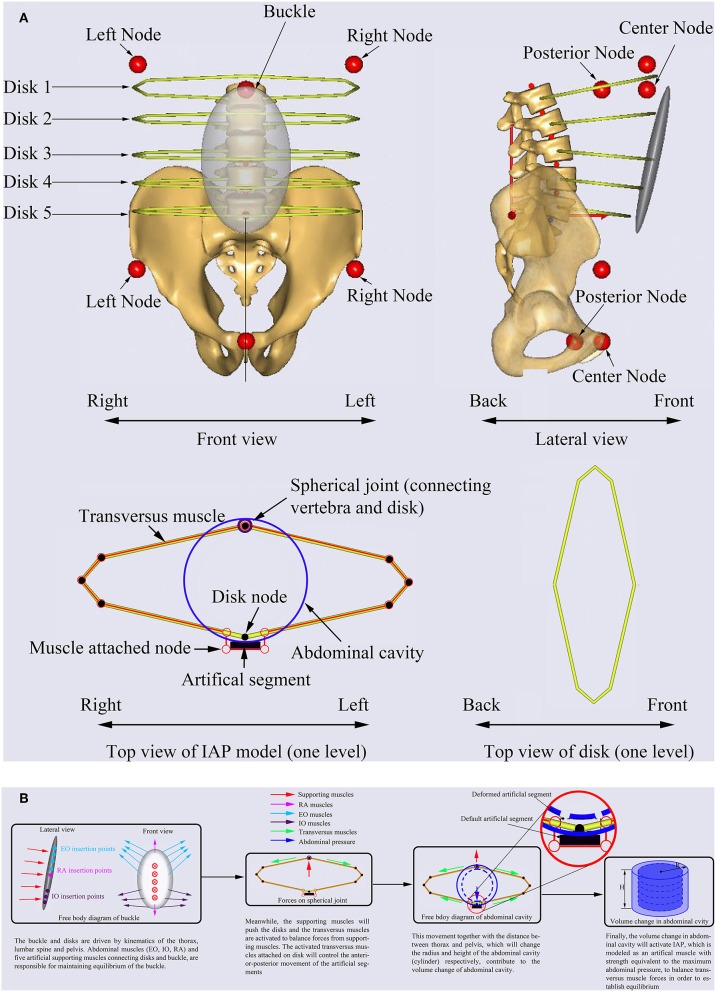
Description of the IAP modeling **(A)** and mechanism of IAP generation **(B)** in AnyBody.

The abdominal cavity was simulated using a cylinder with maximum pressure equal to 26.6 kPa (Essendrop, 2003). The IAP model is mainly composed of one rigid buckle that provides attachments to the abdominal muscles (EO, IO, RA) and five rigid artificial disks forming structure for the transversus muscles which are responsible for generating IAP (Figure 2A). The buckle and artificial disks are driven by kinematics of the thorax, lumbar spine, and pelvis. The abdominal muscles (EO, IO, RA) and five artificial supporting muscles connecting artificial disks and buckle, are responsible for maintaining equilibrium of the buckle (Figure 2B). The supporting muscles push the artificial disks (Figure 2B) which activates the transversus muscles to maintain the equilibrium of the buckle. The activated transversus muscles attached to the artificial disks will control the anterior-posterior movement of the artificial segments (Figure 2B). This movement together with the distance between thorax and pelvis, which will change the radius (R) and height (H) of the abdominal cavity (cylinder), respectively, contribute to the volume change of abdominal cavity, and their relationship can be expressed using Equation (2).

(2) $$\begin{array}{r} V=V_{0}+\overset{5}{\underset{i=1}{\sum }}\frac{dV}{dR_{i}}\Delta R_{i}+\frac{dV}{dH}\Delta H \end{array}$$

where *V*, is the volume of the cylinder, *V*_0_ is the initial volume of the cylinder, R represents the radius of the cylinder at each artificial disk, and H is the height of the cylinder.

Finally, the change in abdominal cavity will activate the IAP, which is modeled as an artificial muscle with strength equivalent to the maximum abdominal pressure, to balance the transversus muscle forces and establish equilibrium (Figure 2B). In other words, any change in these supporting muscles will affect the force in the transversus muscles which in turn will influence the IAP. This pressure will then act on the nodes defined on the thorax and pelvis as concentrated forces (Figure 2A). All muscles used in the model of IAP are governed by the optimization function used for the entire MSK model. The range of IAP values were approximated to vary between 0.1 and 5.7 kPa from neutral standing to forward flexion (60°) (Schultz et al., 1982). The lumbo-pelvic ratio and lumbar rhythm were selected based on published experimental data (Granata and Sanford, 2000; Arjmand and Shirazi-Adl, 2006b). The muscle forces, and joint forces at the T12-L1 junction predicted by the MSK model together with the gravitational forces were input into our previously developed and validated FE model to predict the IDP, disc forces and moments, and spinal load-sharing.

### Finite Element Model

Geometry of the lumbosacral vertebrae (L1-S1) in neutral standing posture was exported from the MSK model to create the FE model after detailed cleaning of spikes and sharp edges using Geomagic software (Geomagic Studio 2014, 3D System, USA). Geometry meshing was conducted using the software Hypermesh (Hyperworks 14.0, Altair, USA). The adjacent endplates were first meshed using 4 node shell element and then extruded to create 7 layers of 8-node brick element to create the intervertebral disc which included annulus fibrosis and nucleus pulposus with volumes equal to 56 and 44% of the disc volume, respectively (Schmidt et al., 2007; El-Rich et al., 2009). Non-linear springs, distributed in concentric lamellae with a crosswise pattern close to ±35°, were used to model the annular fibers (Schmidt et al., 2007; El-Rich et al., 2009). The cortical bones were meshed with 3-node shell element and filled with 4-node solid elements to simulate the cancellous bone. Five pairs of frictionless surface-to-surface contact were created between adjacent facets with a gap of 1.5 mm along L1-S1 levels. In addition, seven types of ligaments were modeled as non-linear springs having the same non-linear behavior and the same insertion and origin points as those of the MSK model and resisting only tension forces. The material properties used in the FE model are summarized in Table 2.

**Table 2:** Element type and material properties of the FE model.

**Spinal components**	**Element type**	**Material behavior**	**Mechanical properties**	**References**
Cortical bone	3-node Shell		E = 12,000 MPa, ν = 0.3	
Cartilaginous endplates	3-node Shell	Linear elastic	E = 23.8 MPa, ν = 0.4	Naserkhaki et al., 2016
Cancellous bone	Tetrahedral		E = 200 MPa, ν = 0.25	
Annulus ground substance	Hexahedral	Hyper-Elastic (Mooney-Rivlin)	C_10_ = 0.18, C_01_ = 0.045	Schmidt et al., 2006
Nucleus pulposus	Hexahedral		C_10_ = 0.12, C_01_ = 0.030	
Collagen fibers	2D spring	Non-linear force-displacement curve		Shirazi-Adl et al., 1986

Five FE models of the L1-S1 functional spinal units devoid of ligaments and facet joints were subjected to pure moments of 7.5 Nm in flexion and extension to predict the flexural stiffness of the intervertebral discs. These non-linear stiffness curves were used in the MSK model to simulate the spherical joints.

The joint forces, ligament forces, facet joint forces (null in both upright and forward flexion postures in this simulation) and muscle forces predicted at the junction T12-L1 together with muscle forces at all spinal levels of the MSK model were applied to the FE model. The resultant reaction force (shear and compression) at T12-L1 joint, however, was substituted by a sagittal translation applied in the direction of the reaction force to correct the small discrepancy between the deformed position predicted by the MSK model and the one resulted from the FE model. This discrepancy is due to the difference in the approaches used to model the disc in both models, and this iteration process was performed until the reaction force generated by sagittal displacements in FE model was almost equal (within predefined tolerance) to its counterpart predicted by the MSK model under the same posture. The gravitational force of each vertebra was also applied to the FE model. The sacrum was tilted according to the lumbo-pelvic rhythm used in the MSK model and then it was fixed throughout simulation.

### Simulated Tasks

Forward flexion (60°) posture was selected to investigate the influence of the IAP on muscle forces, spinal loading and load-sharing. The IAP was activated (IAP_ON) and deactivated (IAP_OFF) by setting the IAP (artificial muscle activity) to normal and zero, respectively (Arshad et al., 2016). During flexion, the arms were always kept parallel to the direction of gravity.

## Results

### IAP

**Figure 3:**
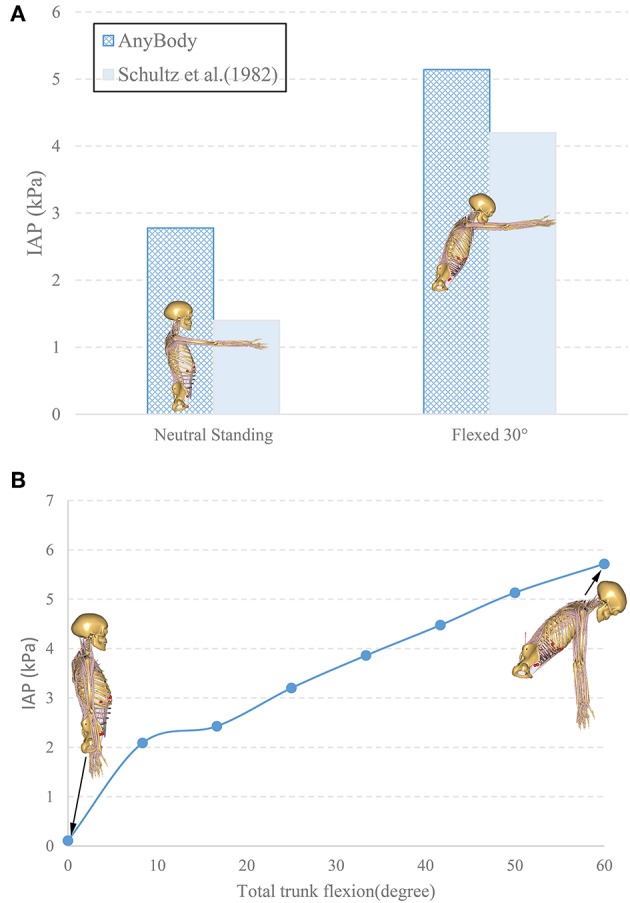
Comparison of the predicted IAP with *in vivo* experimental data (Schultz et al., 1982) **(A)**. The change of IAP in the MSK model from neutral standing to 60° forward flexion **(B)**.

The IAP model in the MSK model was validated by quantitatively comparing the predicted IAP values to *in-vivo* experimental data measured in upright and 30° forward flexion postures with hands raised horizontally in front of the thorax (Figure 3A; Schultz et al., 1982). In agreement with the experimental findings, the model revealed a significant increase in the IAP from the neutral standing posture to 30° forward flexion (Figure 3A). The predicted IAP was 2.7 kPa, which is 1.3 kPa higher than the value reported by Schultz et al. (1982), while in forward flexion posture, the model predicted an IAP of 5.1 kPa, which is 1.3 kPa greater than its counterpart measured experimentally. These discrepancies could result from the inter-individual variability and the differences in the methods used to measure the IAP.

Results of the simulated postures (upright and 60° forward flexion with arms parallel to the gravity direction) revealed an increase of IAP from 0.1 to 5.7 kPa as the trunk flexed during the entire simulation (Figure 3B). The magnitude of 0.1 kPa in the neutral standing posture agreed with its counterpart (0.2 kPa) in literature (Andersson et al., 1976).

### Muscle Force

**Figure 4:**
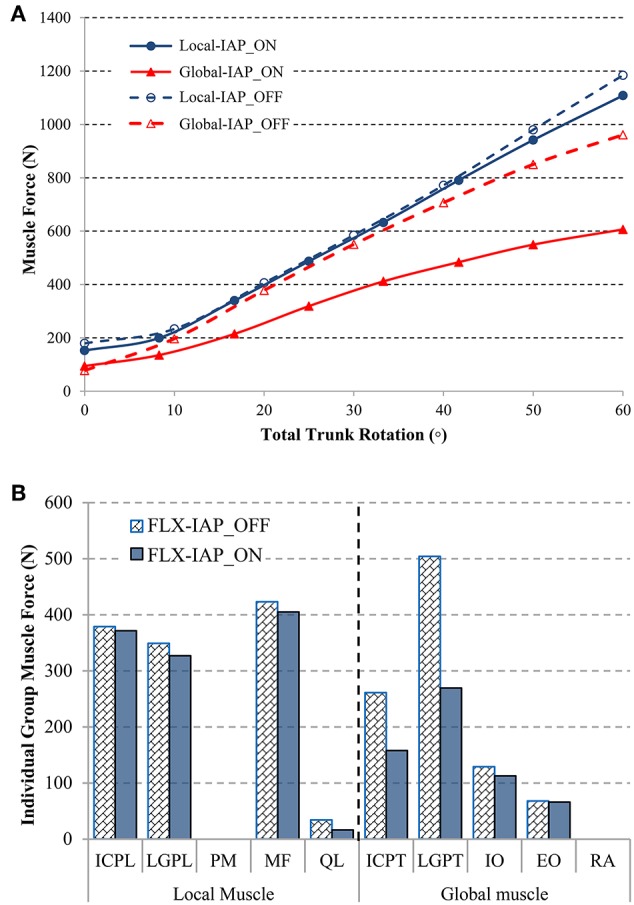
Comparison of the predicted global and local muscle forces under activation (IAP_ON) or deactivation (IAP_OFF) of the IAP during forward flexion **(A)**. Local and global muscle forces at 60° forward flexion for both activated IAP (IAP_ON) and deactivated IAP (IAP_OFF) models **(B)**.

The sum of the global and local muscle forces, with and without the IAP, were predicted using the MSK model (Figure 4A) as the lumbar spine flexion varied from 0 to 60°. In the neutral standing posture, the total local muscle force was predicted at ~179 N, which was 27 N higher than the results from the model without IAP. In contrast, the total global muscle force was 78 N at the same posture, which was 17 N lower as compared with the alternate model settings. Both global and local muscle forces increased substantially with the inclination of the trunk to reach 961 and 1185 N, respectively, when the IAP was excluded. Activation of this latter in the MSK model reduced the total global muscle force substantially along with the inclination of the trunk. This reduction reached 37% at 60° flexion. The total local muscle force decreased as well. However, the reduction started at 40° and reached its maximum value of 6.5% at 60° flexion.

The total force of each individual muscle group was predicted at the maximal trunk inclination (Figure 4B). The pronounced unloading effect of IAP was observed for almost all muscle groups, except for the Psoas Major (PM) and the Rectus Abdominis (RA), which remained silent regardless of the IAP settings.

In the local muscle group, the MF muscle contributed the most at 60° forward flexion, reaching 423 N, followed by the ICPL and LGPL, whose values were 379 and 349 N, respectively. The QL muscle produced the smallest force (34 N). In the global muscle group, the LGPT produced the greatest force (504 N) followed by the ICPT muscle (261N). The force in the abdominal muscles did not exceed 68 and 129 N in the EO and IO muscles, respectively. These values correspond to the case of deactivated IAP. Including IAP in the model did not change the muscle forces pattern. However, it clearly reduced the force in all muscles particularly in the QL muscle and the global extensors LGPT and ICPT where the drop reached 52, 46, and 40%, respectively. The maximum decrease of the force in the remaining extensor and abdominal muscles did not exceed 12%.

### Annular Fiber Strain

High tensile fiber strain was produced at the innermost lamellae at either the posterior or anterior or both regions, except at the L1-2 level, regardless of the existence of IAP. In the presence of the IAP, predicted high tensile strain in the collagen fibers was observed in the anterior region of the innermost lamella at L2-3 level. This high strain was then transferred to the posterior region of the innermost lamella at L3-4 level. High tensile stain in both anterior and posterior regions of the lamella was also observed at the L4-5 level. This trend became more pronounced at the L5-S1 level.

**Figure 5:**
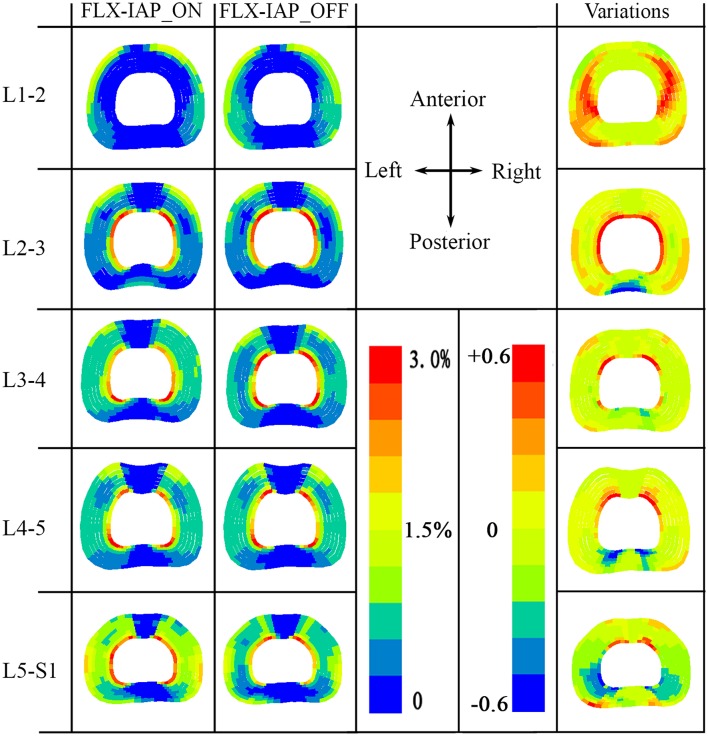
Annular fibers strain at all levels (L1-S1) predicted by the FE model at 60° forward flexion with both IAP settings. Variations were calculated with respect to the case with IAP activated (FLX-IAP_ON).

In contrast, the proportion of high tensile strain increased in the corresponding area of the lamellae for all discs, except at the L5-S1 level in the absence of IAP effects. A noticeable reduced proportion of high tensile strain, however, was produced at the L5-S1 under the same IAP condition (Figure 5).

Variation of the annular tensile strain due to the inclusion or not of IAP (shown on the right end of Figure 5) was calculated as the strain of the model with no IAP minus it counterpart of the model with IAP. The maximum positive variation occurred in the lateral left and right regions of the lamella of disc L1-2, and in the innermost region of the lamella for the remaining levels. The region of maximum variations decreased from upper to lower levels of the spine (Figure 5). The minimum variation corresponding to the case where the model with IAP predicted higher tensile strains, occurred in the posterior outermost region of the lamella at L2-3 level and in the posterior innermost region of the lamella at L3-S1 levels. The area of the minimum variations increased gradually from middle to lower levels of the spine.

### IDP

**Figure 6:**
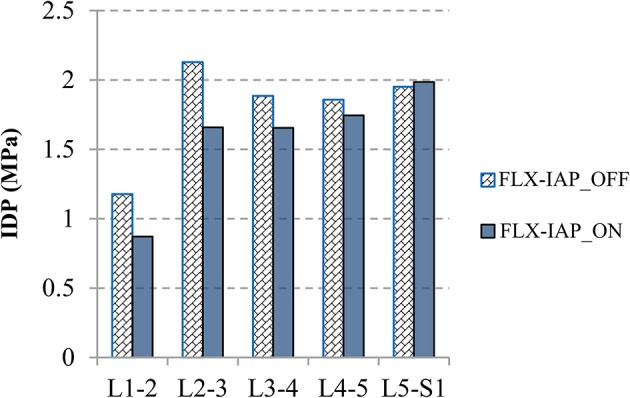
IDP values at all spinal levels predicted at 60° forward flexion angle for both IAP settings.

The IDP was calculated by averaging the pressure in all elements of nucleus (Naserkhaki et al., 2016; Liu et al., 2018) and exhibited the same pattern at all lumbar levels (L1-5) with or without accounting for the IAP (Figure 6). On the other hand, a noticeable decrease in the IDP was observed in the presence of IAP at all levels except the L5-S1 level. The greatest drop occurred at the L1-2 level and reached 26% while the magnitude of IDP remained almost unchanged at the L5-S1 level.

### Disc Forces and Moment

**Figure 7:**
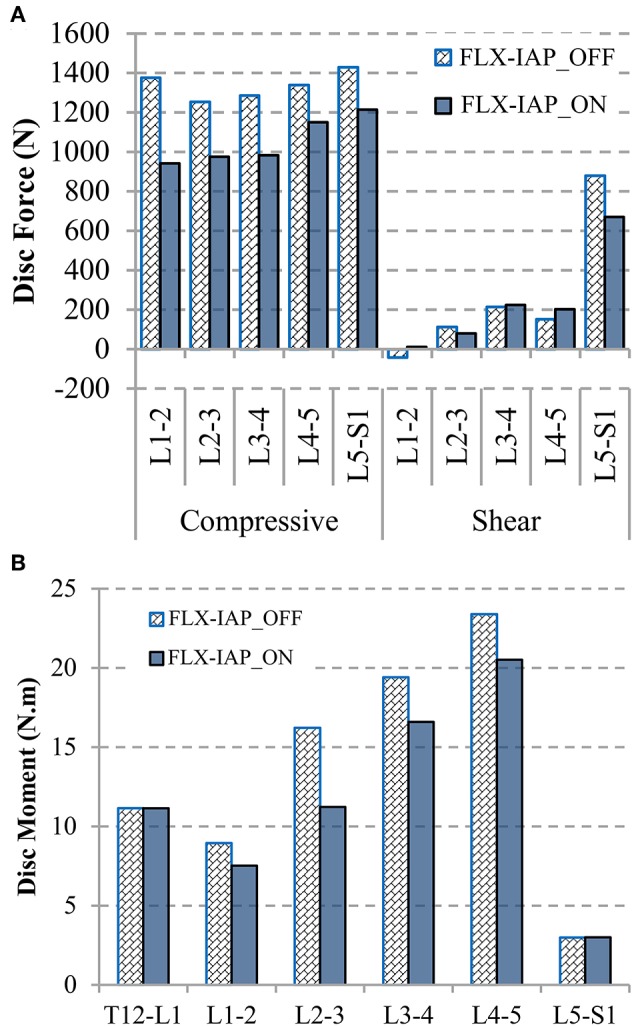
Disc compressive and shear forces (+ve in anterior direction) **(A)** and disc moments (+ve in flexion) **(B)** at 60° forward flexion predicted by the FE model.

The disc compressive force followed the same pattern, a decrease from the L1-2 level to the L2-3 level followed by an increase along the lower levels, in both cases, with and without the IAP. Activating this latter reduced the compressive force at all levels. The decrease ranged from 15 to 32% at the levels L5-S1 and L1-2, respectively. When the IAP was active, the disc shear force reduced by 24 and 28% at the L5-S1 and L2-3 levels, respectively. However, the L3-4 and L4-5 levels experienced an increase of 5 and 33%, respectively and the shear force changed direction from anterior to posterior at the L1-2 level (Figure 7A). The disc moment also dropped along the spinal levels except at the T12-L1 and L5-S1 levels when the IAP was included (Figure 7B). The greatest change was 31% and occurred at the level L2-3.

### Ligaments Forces

**Figure 8:**
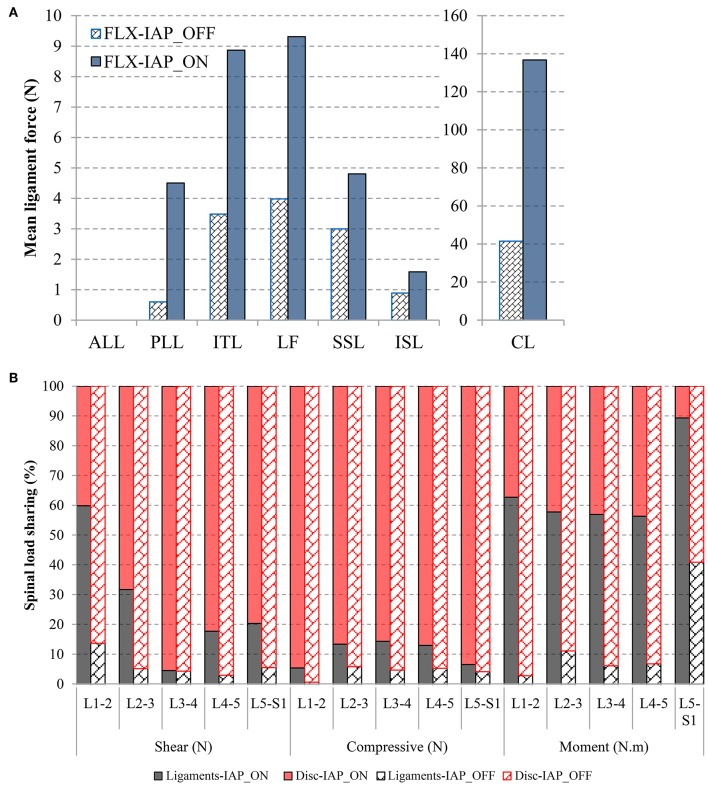
Effects of IAP on ligament force **(A)** and load-sharing of the passive structures (discs and ligaments) **(B)** evaluated at 60° forward flexion. The facet joints have no contribution to load-sharing.

Activating the IAP increased the force in all ligaments significantly. The highest increase was found in the PLL (from 0 to 5 N) and CL (from 40 to 140 N) ligaments (Figure 8A). The ALL ligament experienced zero force in both IAP settings.

### Spinal Load-Sharing

In the absence of IAP, the compressive force was resisted mostly by the disc while the ligament contribution did not exceed 5%. The ligaments had also minor contribution (<14%) to resist shear force and moment as compared to the discs except at the L5-S1 level where they carried about 41% of the moment. The facet joints had no contribution at all to load-sharing (Figure 8B).

Activating the IAP, increased the role of the ligaments in carrying compressive and shear forces, as well as moments. The increase of the ligament contribution to moment resistance was substantial at all spinal levels. For instance, the ligament moment-sharing jumped from 14 to 60% and from 5 to 32% at the L1-2 and L2-3 levels, respectively. The facet joints remained silent in all cases.

## Discussion

Despite the ongoing debate regarding which abdominal muscle is responsible for raising the IAP (Daggfeldt and Thorstensson, 2003; Cholewicki and Reeves, 2004), the role of this latter in unloading and stabilizing the lumbar spine has been established in the past few decades (Daggfeldt and Thorstensson, 1997, 2003; Cholewicki and Reeves, 2004; Arjmand and Shirazi-Adl, 2006a; Stokes et al., 2010; Park et al., 2013), and is well-accepted within the spinal biomechanics community. The influence of the IAP on spinal load-sharing, however, remains not well-studied. This work attempted to quantify these effects during static forward flexion (60°), a posture associated with high abdominal muscle activity (Cresswell and Thorstensson, 1989), using our previously developed and validated method that combines MSK and FE models to predict muscle, ligaments, and discs forces, and moments as well as IDP and spinal load-sharing.

As a submodel of our current MSK model, the IAP was compared with *in-vivo* experimental data, quantitatively presenting an overall good match during neutral standing and forward flexion (Schultz et al., 1982; Figure 3A). In addition, the predicted IAP (Figure 3B) in the neutral standing posture was quite close to its literature counterpart (Andersson et al., 1976). Other experimental data, which have been obtained during valsalva maneuvers or maximum voluntary strength exertions (Nachemson et al., 1986; Cholewicki et al., 1999, 2002), were not compared here since they were not considered as realistic representatives of the IAP role in static postures (Arjmand and Shirazi-Adl, 2006a; Stokes et al., 2010).

In alignment with previous studies (Arjmand and Shirazi-Adl, 2006a; Arshad et al., 2016), our results revealed that the inclusion of the IAP in the MSK model leads to a decrease in muscle forces, which is more pronounced in the global muscle group at larger flexion angles (Figures 3, 4). More specifically, the forces in two global muscle groups: the iliocostalis lumborum pars thoracic (ICPT) and the longissimus thoracis par thoracic (LGPT), decrease substantially in the presence of the IAP. This also confirmed that the IAP could produce an extensor moment, which reduces the activity of the erector spinae muscles and, thus, alleviates spinal loads (Bartelink, 1957; Daggfeldt and Thorstensson, 2003). In addition, such significant decrease confirms the hypothesized unloading role of the IAP and stresses the importance of its incorporation in simulation models of the lumbar spine, particularly when subjected to forward inclination (Cholewicki and Reeves, 2004). The unloading role of the IAP in flexion can also be confirmed by the predicted disc force and moment. In the presence of the IAP, the compressive force decreases up to 434 N (31%) at all levels, while a maximum reduction of 208 N (24%) in the shear force occurs. A maximum decrease up to 5 N.m (32%) in disc moments at the L1-5 levels is also found, which is in agreement with previous work (Daggfeldt and Thorstensson, 2003). The reduction in the disc loads due to activating the IAP is compensated for by an increase in ligaments forces to maintain the equilibrium at the same deformed posture, i.e., under similar loading conditions. This confirms that neglecting the IAP in spine biomechanics studies would underestimate the role of the ligaments and potentially yield unrealistic predictions of disc forces and moments.

The variations among the annular fibers strain between the two cases studied (IAP_ON and IAP-OFF) were little. A small increase in the proportion of the high tensile strain fibers was observed at the L1-4 levels in the model with no IAP, which is mainly due to the increase in the muscle (global and local) forces applied to the FE model, which in turn increased the IDP.

It is noteworthy that the IDP decreased at all levels except the L5-S1 level, which confirms again the previously mentioned hypothesized unloading role of the IAP. An increase up to 0.5 MPa in the IDP was observed at the L2-3 level without consideration of IAP effects. The reduction of the IDP was smaller at the lower levels L3-5, in agreement with Hodges et al. (2005) who found that the IAP has more effects on the L2 vertebra as compared to the L4.

Load distribution among the various passive components is markedly altered in the presence of the IAP. Our results confirmed that the main contribution of the disc is to resist external load in forward flexion, which is more pronounced without IAP simulation. The disc force- and moment-sharing varied between 86 and 100% of the total spinal force and moment, except at the L5-S1 level, where the ligaments moment-sharing reached 40%. Including the IAP alleviated the disc load and increased the ligament load-sharing, particularly the moment sharing.

### Model Assumptions and Limitations

The current MSK model predicted the IAP based on the change of the abdominal cavity volume during forward flexion, rather than using typical prescribed experimental IAP values available in literature (Cholewicki et al., 1999; Arjmand and Shirazi-Adl, 2006a; Stokes et al., 2011). The model also considered the interaction between abdominal muscles, physiological cross section area and strength of these muscles. The transversus muscle, considered as a significant contributor to the rise in the IAP (Cresswell et al., 1992; Cresswell, 1993), was also included in the IAP model. Setting the IAP (artificial muscle activity) to zero (Arshad et al., 2016) in order to switch it off in Anybody did not eliminate the force in the abdominal muscles (EO and IO), as these muscles are attached to the buckle and artificial disks and contribute to their equilibrium (Figure 2B). Similar kinematics were considered in both IAP settings, and no co-activity antagonism was simulated in this study. Although it is established that trunk stability is intimately associated with the elevated IAP, this was not taken into consideration in the current study. This is due to the fact that daily flexion is regarded as a skill posture (de Zee et al., 2007), which has been widely investigated using optimization models (El-Rich et al., 2004; Arjmand and Shirazi-Adl, 2005, 2006a; Stokes et al., 2010; Park et al., 2013). By minimizing the overall muscle stress, activation of muscles and spinal loads may have been underestimated as compared with realistic loads. Had the activation of muscle pattern changed, the effects of the IAP would have need to be re-evaluated (Arjmand and Shirazi-Adl, 2006a). Other limitations related to methodology are mentioned elsewhere (Liu et al., 2018).

## Conclusions

In summary, the current research investigated the influence of the IAP on muscle forces, loads in the passive spinal structures, as well as load-sharing during forward flexion using a previously validated tool that combines a MSK of the upper body and a FE model of the lumbosacral spine. In alignment with literature, this study confirmed the unloading role of the IAP during upper body inclination. The IAP had significant influence on global muscle forces, yet, negligible effects on local muscle forces. The substantial increase in the IDP, internal disc force and load sharing, triggered by absence of the IAP, should be taken into consideration in future modeling efforts of the lumbar spine in flexion postures. This is the first study to the best knowledge of the investigators that attempts to quantitatively assess the role of the IAP on detailed spinal biomechanics. Such information is essential for the accurate modeling of the spine toward more effective therapeutic and rehabilitative modalities, as well as the design and development of artificial implants.

## Data Availability Statement

All datasets generated for this study are included in the manuscript.

## Author Contributions

TL simulated all the models in AnyBody and applied the corresponding boundary conditions from AnyBody to finite element model. He also drafted the manuscript and contributed to the interpretation of the results. KK contributed greatly to the improvement of the manuscript and provided the technical support for the analysis in AnyBody. SA gave substantial help with FE modeling and provided valuable insights in the improvement of the paper. ME-R was the supervisory author and contributed to the manuscript composition, and the conception and design of the work. All the authors made substantial contributions to this study and approved to publish the manuscript in its current version.

### Conflict of Interest

The authors declare that the research was conducted in the absence of any commercial or financial relationships that could be construed as a potential conflict of interest.
